# Effects of COVID-19 on Mental Health and Anxiety of Adolescents Aged 13–16 Years: A Comparative Analysis of Longitudinal Data From China

**DOI:** 10.3389/fpsyt.2021.695556

**Published:** 2021-07-20

**Authors:** Jie Qin, Yueyue Ding, Jing Gao, Yun Wu, Haitao Lv, Jian Wu

**Affiliations:** ^1^Department of Pediatrics, The Fourth Affiliated Hospital of Nantong University, Yancheng City, China; ^2^Department of Pediatrics, The First People's Hospital of Yancheng, Yancheng City, China; ^3^Department of Cardiology, Children's Hospital of Soochow University, Suzhou, China; ^4^Junior High, Suzhou International Academy, Beijing Foreign Studies University (BFSU), Suzhou, China; ^5^Yancheng Primary School, Yancheng, China; ^6^Department of Clinical Laboratory, The Affiliated Suzhou Hospital of Nanjing Medical University, Suzhou Municipal Hospital, Gusu School, Nanjing Medical University, Suzhou, China

**Keywords:** COVID-19, adolescents, mental health, psychosocial problems, anxiety

## Abstract

**Background:** Adolescence is an important stage of psychological development, and the psychological and mental problems of many adults are affected by the COVID-19 epidemic. The aim of this study was to understand the psychological status of this group during the epidemic, and to determine the risk factors leading to psychological stress, as well as protective factors.

**Methods:** An online survey was run on April 2, 2020. The participants were 254 adolescents aged 13–16 years from a junior high school in Jiangsu, China. The results were compared with the pre-epidemic data, which came from the psychological status survey routinely carried out by the school. Mental health variables were assessed via the Mental Health Test that included one validity subscale and eight content subscales.

**Results:** The number of adolescents with poor mental health increased significantly from 12.3 to 24.2%. There was significant increase in learning anxiety (33.7 vs. 56.4%), sensitivity tendency (19.8 vs. 46%), somatic anxiety (13.9 vs. 40.7%) and phobia tendency (4.4 vs. 10.1%). During the epidemic, there were significant differences between adolescents with normal and poor mental health in family structure, personality, relationship with siblings, daily exercise time, and risk of family members coming in contact with COVID-19. Living in stem family, no siblings, and risk of contracting COVID-19 from family members were significant risk factors for teenagers with poor mental health. Risk of contracting COVID-19 from family members was the most influential risk factor for learning anxiety, self-blaming tendency, sensitivity tendency, and somatic anxiety. Exercising for ≥1 h per day was a significant protective factor for poor mental health.

**Conclusions:** During the COVID-19 epidemic, adolescents aged 13–16 years have had psychosocial problems, especially learning anxiety, sensitivity tendency, somatic anxiety, and phobia tendency, as well as risk factors for developing them. Our study provides insights for potential interventions.

## Introduction

The epidemic of the coronavirus disease 2019 (COVID-19) constitutes a public health emergency of international concern, and poses a major threat to human life. As of April 30, 2020, >4.5 million people worldwide have fallen ill and >2,900,000 have died (1). The extent and impact of COVID-19 far exceed those of severe acute respiratory syndrome (SARS) (2). Frontline healthcare professionals and infected patients have always been the groups of most concern, but concern has been extended to the general population, including the psychological effects of coronavirus on anxiety, depression, helplessness and stigma (3–5). Fortunately, the morbidity of adolescents has been low during the epidemic; however, that does not mean that they have not been affected. Colizzi et al. reported a case of symptom exacerbation of a severe and persistent somatic, triggered by the fear of having COVID-19, and after treatment with the antipsychotic, dietary counseling, psychological support, the symptoms were significantly improved (6).

Adolescence is an important stage of psychological development, and the psychological and mental problems of many adults are affected by this period (7, 8). The core risks of this period are the development of symptoms and syndromes of anxiety that may range from transient mild symptoms to full-blown anxiety disorders. For adolescents between the ages of 13 and 16 years, the COVID-19 epidemic is a huge source of stress, similar to that of the SARS epidemic (9), which they have not previously encountered. Almost all of them have been isolated at home, with a small number of patients, and some are close contacts of patients, suspected patients or medical workers. Therefore, the direct and indirect psychological impact of the epidemic cannot be ignored. However, few studies have investigated the psychological impact of COVID-19 on adolescents aged 13–16 years in China.

In this study, we wanted to understand the psychological status of this group during the epidemic, and determine the risk factors leading to psychological stress, as well as protective factors, in order to provide insights for potential interventions.

## Materials and Methods

### Participants and Procedure

We carried out this study in the junior middle school of a foreign language school in Jiangsu, China, which is a boarding school and the students' families were in good economic condition. The students live at school 5 days a week, with four people in a room and better living facilities. These teenagers have an hour a day to talk to their parents. The weekend is a rest day, and the children go back to their homes to get together with their families. The surveys were completed in an online classroom on April 2, 2020. Students aged 13–16 years participated in the surveys with the consent of their parents and themselves. None of them had major physical and psychiatric morbidities. The survey was completed in the form of an electronic questionnaire within 40 min under the video guidance of the school psychology teacher. Children who had difficulty on the Internet or asked for leave did not participate in the survey. The participants filled in some information about demographic data. A total of 254 questionnaires were distributed and collected, of which 248 were valid, and 6 questionnaires were excluded because of the high score of the validity scale. This project was approved by the Ethics Committee of Yancheng No. 1 People's Hospital (No. 2020K027). The participants were not paid and their parents were enthusiastic about the assessment of their children's psychological status.

The school pays great attention to the mental health of its students. In October every year, the psychology teacher conducts a questionnaire survey of the children. On October 12, 2019, the same group of adolescents as in the present study answered the same questionnaire in the psychology class. A total of 260 papers were distributed and collected, of which 8 were not reliable. These data provide us with the situation before the epidemic, but unfortunately, the questionnaire was anonymous and we did not have demographic information at that time.

### Measurements

The following demographic data were collected: sex; age; living in urban or rural area; family structure (stem family—a family of three generations, nuclear family-parents and children only, extended family—parents and several pairs of married children, and single parent family); personality (introvert or extrovert); presence or absence of siblings; risk of family members coming in contact with COVID-19 (family members were medical workers, volunteers, community workers, or others with a high risk of contact with COVID-19); exercise time per day (<1 or ≥1 h); and number of times per week that participants left home during quarantine (never, 1 or 2 and ≥3 times).

We used the Mental Health Test (MHT), adapted by Professor Zhou Bucheng, East China Normal University, based on the General Anxiety Test, which is a popular adolescent anxiety scale in China (10, 11). These tests are variants of the Children's Manifest Anxiety Scale (CMAS). CMAS is widely used to measure children's anxiety in Europe and North America and is an internationally recognized standardized scale (12).

There were 100 items that needed to be answered in MHT, and respondents were asked to choose “yes” (score 1) or “no” (score 0). The scale contained 8 subscales and one validity scale. Eight subscales scales contained 90 items that measured eight specific subcategories of anxiety: learning anxiety, personal anxiety, loneliness anxiety, self-blaming tendency, sensitivity tendency, somatic anxiety, phobia anxiety, and impulsive tendency. Any subcategory score >8 meant clinical warning and indicated that the respondent was more likely to need further assessment, even psychological treatment. Ten items constitute the validity scale. The score of the validity scale represents the authenticity, and the questionnaire with more than 8 points indicates poor credibility and should be removed. The total score of the respondents was used to assess their mental health. The higher the score, the more serious the psychological anxiety, and a score ≥56 was considered to represent poor psychological status, including psychological problems. The test–retest reliability of MHT ranges from 0.67 to 0.86, and the correlation between each individual category and the total score measures 0.52–0.7 (13, 14).

### Statistical Analysis

χ^2^-tests were used to compare group differences of categorical data. Multivariate logistic regression was performed using stepwise variable selection, and all variables were entered into the model to explore independent influence for different risk dimensions, such as learning anxiety, personal anxiety, loneliness anxiety, self-blaming tendency, sensitivity tendency, somatic anxiety, phobia anxiety, and impulsive tendency. All hypotheses were tested at a significance level of 0.05. Data analyses were run on SPSS version 23.0.

## Results

### Psychological Manifestations of Adolescents Before and During the COVID-19 Epidemic

Table 1 presents the psychological changes in this group of adolescents before and during the COVID-19 epidemic. Compared with before the epidemic, the proportion of adolescents with poor psychological status during the epidemic increased from 12.3 to 24.2% (*P* = 0.001). There were significant changes in learning anxiety (33.7 vs. 56.4%, *P* < 0.01), sensitivity tendency (19.8 vs. 46%, *P* < 0.01), somatic anxiety (13.9 vs. 40.7%, *P* < 0.01), and phobia tendency (4.4 vs. 10.1%, *P* = 0.01). Personal anxiety, loneliness anxiety, self-blaming tendency, and impulsive tendency were no different from those before the epidemic.

**Table 1:** Psychological manifestations of adolescents before and during the COVID-19 epidemic.

**Characteristics**	**Before the epidemic**	**During the epidemic**	***P***
	**(*n* = 252)**	**(*n* = 248)**	
**General MHT, % (*****n*****)**			0.001
≤55 points	87.7 (221)	75.8 (188)	
>55 points	12.3 (31)	24.2 (60)	
**Learning anxiety, % (*****n*****)**			<0.01
≤8 points	66.3 (167)	43.5 (108)	
>8 points	33.7 (85)	56.4 (140)	
**Personal anxiety, % (*****n*****)**			0.21
≤8 points	91.7 (231)	86.9 (219)	
>8 points	8.3 (21)	11.5 (29)	
**Loneliness anxiety, % (*****n*****)**			0.83
≤8 points	90.5 (228)	89.9 (223)	
>8 points	9.5 (24)	10.1 (25)	
**Self-blaming tendency, % (*****n*****)**			0.93
≤8 points	80.2 (202)	79.8 (198)	
>8 points	19.8 (50)	20.6 (50)	
**Sensitivity tendency, % (*****n*****)**			<0.01
≤8 points	80.2 (202)	54.0 (134)	
>8 points	19.8 (50)	46.0 (114)	
**Somatic anxiety, % (*****n*****)**			<0.01
≤8 points	86.1 (217)	59.3 (147)	
>8 points	13.9 (35)	40.7 (101)	
**Phobia anxiety, % (*****n*****)**			0.01
≤8 points	95.6 (241)	89.9 (223)	
>8 points	4.4 (110)	10.1 (25)	
**Impulsive tendency, % (*****n*****)**			0.16
≤8 points	94.0 (237)	90.7 (225)	
>8 points	61.0 (5)	9.3 (230)	

### Comparison of Sociodemographic Features Between Normal and Poor Mental Health Groups During the COVID-19 Epidemic

Table 2 presents sociodemographic features of the adolescents during the COVID-19 epidemic and compared the normal mental health group (*n* = 188) with the poor mental health group (*n* = 60). There were significant differences in family structure, personality, siblings, exercise time per day, and family members at risk of coming in contact with COVID-19 between adolescents with normal and poor mental health. The adolescents with normal mental health were more likely to come from nuclear families (81.9 vs. 51.7%, *P* < 0.01), have extrovert personality (67 vs. 45%, *P* < 0.01), have siblings (55.3 vs. 40%, *P* = 0.04), exercise ≥1 h/day (33 vs. 10%, *P* < 0.01), and have no risk of family members coming in contact with COVID-19 (90 vs. 56.7%, *P* < 0.01). There was no significant difference in sex, living area and number of times the respondents left their home per week during the epidemic.

**Table 2:** Comparison of sociodemographic features between adolescents with normal and poor mental health during the COVID-19 epidemic.

**Characteristics**	**Normal mental health**	**Poor mental health**	***P***
	**(*n* = 188)**	**(*n* = 60)**	
**Sex, % (*****n*****)**			0.17
Male	48.4 (91)	38.3 (23)	
Female	51.6 (97)	61.7 (37)	
**Age (years), % (*****n*****)**			0.67
13	20.7 (39)	20 (12)	
14	30.3 (57)	31.7 (19)	
15	36.2 (68)	30.0 (18)	
16	12.8 (24)	18.3 (11)	
**Living areas, % (*****n*****)**			0.64
Rural	34.6 (65)	38.3 (23)	
Urban	65.4 (123)	61.7 (37)	
**Family structure, % (*****n*****)**			
Nuclear family	81.9 (154)	51.7 (31)	<0.01
Stem family	18.1 (34)	48.3 (29)	
Extended family	0	0	
Others	0	0	
**Personality, % (*****n*****)**			<0.01
Introverted	33.0 (62)	55.0 (33)	
Extroverted	67.0 (126)	45.0 (27)	
**Siblings, % (*****n*****)**			0.04
No	55.3 (104)	40.0 (24)	
Yes	44.7 (84)	60.0 (36)	
**Exercise time per day,** ** % (*****n*****)**			0.001
<1 h	67.0 (126)	90.0 (54)	
≥1 h	33.0 (62)	10.0 (6)	
**No. of times leaving home, % (*****n*****)**			0.74
0	75.0 (141)	76.7 (46)	
1 or 2	18.6 (35)	15.0 (9)	
≥3	6.4 (12)	8.3 (5)	
**Risk of family members coming in contact with COVID-19, % (*****n*****)**			<0.01
No	90.0 (169)	56.7 (34)	
Yes	10.0 (19)	43.3 (26)	

### Outcomes of Psychological Manifestations

Multivariate logistic regression analyses showed that living in stem family [odds ratio (OR), 3.74; 95% confidence interval (CI), 1.83–7.63; *P* < 0.01], no siblings (OR, 2.21; 95%CI, 1.09–4.49; *P* = 0.03), risk of family members coming in contact with COVID-19 (OR, 6.38; 95% CI, 2.85–14.26; *P* < 0.01) were risk factors for poor mental health, and exercising for ≥1 h per day (OR, 0.23; 95% CI, 0.09–0.62; *P* < 0.01) was a protective factor (Table 3).

**Table 3:** Outcomes of psychological manifestations.

**Variables**	**OR (95%CI)**	***P***
**Models for general MHT**		
Family structure (stem vs. nuclear)	3.74 (1.83–7.63)	<0.01
Siblings (yes vs. no)	2.21 (1.09–4.49)	0.03
Exercise time per day (≥1 vs. <1 h)	0.23 (0.09–0.62)	<0.01
Risk of family members coming in contact with COVID-19 (yes vs. no)	6.38 (2.85–14.26)	<0.01
**Models for learning anxiety**		
Exercise time per day (≥1 vs. <1 h)	0.52 (0.29–0.93)	0.03
Risk of family members coming in contact with COVID-19 (yes vs. no)	2.28 (1.07–4.85)	0.03
**Models for personal anxiety**		
Personality (extrovert vs. introvert)	0.27 (0.11–0.66)	<0.01
**Models for loneliness anxiety**		
Family structure (stem vs. nuclear)	3.14 (1.28–7.70)	0.01
**Models for self-blaming tendency**		
Family structure (stem vs. nuclear)	2.13 (1.04–4.37)	0.04
Daily exercise (yes vs. no)	2.62 (1.28–5.38)	0.01
Risk of family members coming in contact with COVID-19 (yes vs. no)	4.97 (2.26–10.81)	<0.01
**Models for sensitivity tendency**		
Sex (female vs. male)	2.18 (1.25–3.80)	0.01
Daily exercise (yes vs. no)	2.27 (1.30–3.96)	<0.01
Risk of family members coming in contact with COVID-19 (yes vs. no)	4.36 (1.99–9.55)	<0.01
**Models for somatic anxiety**		
Risk of family members coming in contact with COVID-19 (yes vs. no)	9.07 (3.95–20.82)	<0.01
**Models for phobia anxiety**		
Sex (female vs. male)	2.75 (1.02–7.41)	<0.05
Family structure (stem vs. nuclear)	4.08 (1.63–10.20)	<0.01
Exercise time per day (≥1 vs. <1 h)	0.10 (0.01–0.77)	0.03
**Models for impulsive tendency**		
No variables were entered		

*OR, Odds ratio; CI, confidence interval*.

The risk factors for each subscale were different. Risk of family members coming in contact with COVID-19 (OR, 2.28; 95% CI, 1.07–4.85; *P* = 0.03) was independently associated with risk of learning anxiety among adolescents, while exercising for ≥1 h per day was a protective factor (OR, 0.52; 95% CI, 0.29–0.93; *P* = 0.03). For personal anxiety models, extrovert personality (OR, 0.27; 95% CI, 0.11–0.66; *P* < 0.01) was a protective factor. For loneliness anxiety, living in a stem family was an independent risk factor (OR, 3.14; 95% CI, 1.28–7.70; *P* = 0.01). Three variables were independently associated with risk of self-blaming tendency: living in a stem family (OR, 2.13; 95% CI, 1.04–4.37; *P* = 0.04); no siblings (OR, 2.62; 95% CI, 1.28–5.38; *P* = 0.01); and risk of family members coming in contact with COVID-19 (OR, 4.97; 95% CI, 2.26–10.81; *P* < 0.01). There were also three risk factors for sensitivity tendency: female sex (OR, 2.18; 95% CI, 1.25–3.80; *P* = 0.01); no siblings (OR, 2.27; 95% CI, 1.30–3.96; *P* < 0.01); and risk of family members coming in contact with COVID-19 (OR, 6.38; 95% CI, 1.99–9.55; *P* < 0.01). Regarding somatic anxiety, the risk factor was risk of family members coming in contact with COVID-19 (OR, 9.07; 95% CI, 3.95–20.82; *P* < 0.01). For phobia anxiety, risk factors were female sex (OR, 2.75; 95% CI, 1.02–7.41; *P* < 0.05); and living in a stem family (OR, 4.08; 95% CI, 1.63–10.20; *P* < 0.05). Exercising for ≥1 h per day was a protective factor (OR, 0.10; 95% CI, 0.01–0.77; *P* < 0.05). For impulsive tendency, no variable was entered.

## Discussion

The mental health of adolescents aged 13–16 years has been greatly affected during the COVID-19 epidemic, and the number of people who need consultation has increased. In particular, the numbers of adolescents with early signs of learning anxiety, sensitivity tendency, somatic anxiety, and phobia anxiety have increased significantly. In the public health crisis, the risk perception of disease has a negative impact on people's mental health (15). Although adolescents aged 13–16 years are not the main group with COVID-19 virus infection, they must cope with psychological distress and are at risk of allostatic overload (16). Indeed, according to clinimetric criteria, allostatic overload can be diagnosed in the presence of a current identifiable source of distress in the form of recent life events and/or chronic stress; the stressor is judged to tax or exceed the individual coping skills when its full nature and full circumstances are evaluated. The reasons for the psychological distress to which adolescents aged 13–16 years were exposed might be related to many factors, such as being quarantined at home for a long time, facing waves of negative news, fearing that they or their loved ones could be infected by the virus, lack of awareness of the disease, bemoaning the fragility of life, becoming sensitive to their own physical discomfort, and even fear of death. Such distress was seen with the SARS and Ebola virus outbreaks (17, 18).

In this study, the changes in sensitivity tendency, physical anxiety, and phobia anxiety may have been closely related to intolerance of uncertainty. Wright *et al*. explained the relationship between intolerance of uncertainty and adolescent health anxiety (19). Learning anxiety has always been one of the major problems in adolescent mental health that becomes more serious during epidemics (11, 20). This change may be related to the maladjustment caused by the change in learning method from classroom to online teaching.

Generally, the stressors faced by adolescents are their studies, interpersonal relationships, and parents' expectations. However, the COVID-19 epidemic, as an acute infectious disease, has acute, large-scale, and uncontrollable stressors, which is in sharp contrast to the stressors of ordinary life. The relationship between sex, age, place of residence, and mental health has been weakened.

In this study, we found several potential risk factors for adolescents to develop poor mental health, such as family structure, personality, number of siblings, and sex. Undoubtedly, these risk factors might endure allostatic overload and favor the development of psychopathology, including anxiety (20–23). The risk of family members coming in contact with COVID-19 has become the most widespread factor, and has come from awareness of the virus, through the Internet, media, and parents. Intensive media broadcasts, false reports and disinformation about the virus, as well as some extreme case reports, have brought unfounded fears to adolescents. Fear of getting COVID-19, were associated with more negative feelings (24). In addition, negative emotions such as anxiety, fear, tension, and worry spread via social networks (25). As Commodari *et al*. believes, in the face of this unknown virus, appropriate psychological education intervention is very necessary (26). For adolescents, teachers, and parents should pay attention to these and help them to adjust these excessive negative emotions (24, 26, 27). Fortunately, exercising time ≥1 h/day was a protective factor for poor mental health. Other studies have shown that exercise, especially aerobic exercise, can relieve anxiety (28, 29), while sedentary behavior has the opposite effect (30). Hence, physical exercise should be promoted during the epidemic.

The present study had some limitations. First, we adopted a longitudinal design but we did not analyze the psychological changes among different subgroups and the causes for these changes, and the difference between the two questionnaires on social demography limited our conclusion. Second, psychological assessment was based on an online survey and on self-report tools. The use of clinical interviews is encouraged in future studies to allow a more comprehensive assessment of the problem. Third, young people from poor families were not included in our study, and we need to cover a larger sample size and a wider range of socioeconomic groups to make the conclusions more representative.

## Conclusions

During the COVID-19 epidemic, adolescents aged 13–16 years have had psychosocial problems, especially learning anxiety, sensitivity tendency, somatic anxiety, and phobia tendency, as well as risk factors for developing them. They are in need of mental health care and recovery programs. At the same time, our study provides insights for potential interventions. Strengthening physical exercise can alleviate psychological anxiety. Teachers and educators should encourage young people to develop appropriate physical exercises and guide young people's psychology to maintain a healthy and positive mental state. Furthermore, how to guide them is the focus of the next step.

## Data Availability Statement

The original contributions presented in the study are included in the article/Supplementary Material, further inquiries can be directed to the corresponding author/s.

## Ethics Statement

The studies involving human participants were reviewed and approved by this project was approved by the Ethics Committee of Yancheng No. 1 People's Hospital (No. 2020K027), and we obtained informed consent from all students and one of their parents or legal guardians. Written informed consent to participate in this study was provided by the participants' legal guardian/next of kin. Written informed consent was obtained from the individual(s) for the publication of any potentially identifiable images or data included in this article.

## Author Contributions

JQ and YD conceived and designed the study. HL and YW collected data. JW performed the statistical analysis. HL and JQ drafted the manuscript. JW and HL reviewed and revised manuscript. All authors read and approved the final manuscript and content of the final manuscript.

## Conflict of Interest

The authors declare that the research was conducted in the absence of any commercial or financial relationships that could be construed as a potential conflict of interest.

## Abbreviations

COVID-19: coronavirus disease 2019
SARS: severe acute respiratory syndrome
MHT: Mental Health Test
CMAS: Children's Manifest Anxiety Scale
OR: odds ratios
CI: confidence interval.
